# Clinical Outcomes of Platelet-Rich Fibrin in Endodontic Microsurgery: A Systematic Review and Meta-Analysis

**DOI:** 10.3390/jcm15166392

**Published:** 2026-08-18

**Authors:** Carlos Valdivieso del Pueblo, Paloma Montero-Miralles, María León-López, Juan J. Segura-Sampedro, Daniel Cabanillas-Balsera, Jenifer Martín-González, Juan J. Segura-Egea

**Affiliations:** 1 Department of Stomatology, Endodontic Division, School of Dentistry, University of Sevilla, C/Avicena s/n, 41009 Sevilla, Spain; carlos_odn@hotmail.com (C.V.d.P.); mleon12@us.es (M.L.-L.); dcabanillas@us.es (D.C.-B.); jmartin30@us.es (J.M.-G.); 2 Faculty of Health Sciences and Sports, University Center San Isidoro affiliated with Pablo de Olavide University, 41092 Sevilla, Spain; 3 Faculty of Medicine, Universidad San Pablo-CEU, 28040 Madrid, Spain; juan.segura1@ceu.es; 4 Translational Research and Innovation Group in General and Digestive Surgery, Research Institute, Hospital Universitario La Paz (IdIPaz), 28046 Madrid, Spain

**Keywords:** platelet-rich fibrin, endodontic microsurgery, apical surgery, bone regeneration, CBCT, postoperative pain, systematic review

## Abstract

**Background/Objectives:** Platelet-rich fibrin (PRF) and its derivatives have been proposed as biological adjuncts to enhance tissue healing following endodontic microsurgery (EMS). However, current evidence regarding their effects on postoperative recovery and long-term bone regeneration remains inconsistent. This study aimed to evaluate the efficacy of PRF and its derivatives as adjuncts in endodontic microsurgery by separately assessing patient-centered postoperative outcomes and radiographic and volumetric bone-healing outcomes through a systematic review and meta-analysis. **Methods:** This systematic review and meta-analysis was conducted according to PRISMA 2020 guidelines and prospectively registered in PROSPERO (CRD420261400163). Randomized controlled trials and comparative clinical studies evaluating PRF or its derivatives as adjuncts to EMS were eligible. MEDLINE/PubMed, Embase, Scopus, Web of Science, Cochrane CENTRAL, and reference lists were searched from inception to April 2026. Outcomes included postoperative pain, edema, quality of life, clinical–radiographic healing, CBCT volumetric bone regeneration, bone density, and outcomes in complex defects. Risk of bias was assessed using RoB 2 and ROBINS-I. Random-effects meta-analyses were performed where appropriate, and certainty of evidence was evaluated using the GRADE approach. **Results:** Seventeen clinical reports corresponding to sixteen independent datasets were included. PRF and its derivatives improved early postoperative recovery, particularly postoperative pain during the first postoperative week. Meta-analysis demonstrated a significant reduction in early postoperative pain favoring PRF-based interventions (SMD/Hedges g = −0.62; 95% CI, −1.03 to −0.21; *p* = 0.003). Evidence regarding long-term bone regeneration and clinical–radiographic healing remained heterogeneous and inconclusive. CBCT-based studies suggested accelerated early bone healing, although no consistent superiority was demonstrated at 6–12 months. The certainty of evidence ranged from moderate for postoperative pain to very low for volumetric bone regeneration. Interpretation of the findings is limited by clinical and methodological heterogeneity and the small number of studies available for several outcomes. **Conclusions:** The available evidence suggests that PRF may improve early postoperative recovery and soft-tissue healing after endodontic microsurgery. However, current evidence does not support PRF as a predictable substitute for bone grafts or regenerative biomaterials in large or non-contained defects. Well-designed CBCT-based randomized clinical trials with standardized outcome measures are needed.

## 1. Introduction

Apical periodontitis is an inflammatory disease of infectious origin caused by the interaction between microorganisms within the root canal system, their metabolites, and the host immune–inflammatory response. Its progression involves destruction of the periodontal ligament, cementum, and periapical bone, and therefore the primary objective of endodontic treatment is to eliminate infection, resolve inflammation, and preserve the tooth in function [1,2]. When orthograde treatment or nonsurgical retreatment is impossible, unpredictable, or unsuccessful [3], periapical surgery becomes a therapeutic alternative intended to remove pathological tissues, resect the root apex, prepare and seal the root-end cavity, and create favorable conditions for periapical healing [4,5].

Endodontic microsurgery (EMS) has undergone remarkable refinement over the past two decades owing to advances in magnification, illumination, ultrasonic retropreparation, microsurgical instrumentation, and the development of bioceramic retrofilling materials [6,7,8]. These innovations have significantly improved success rates compared with traditional apical surgery [9]. Nevertheless, healing after EMS remains biologically demanding, particularly in cases involving large periapical lesions, cortical plate perforations, through-and-through defects, or combined apicomarginal defects [10,11]. The final healing response may culminate either in fibrous scar tissue formation or in true bone regeneration, depending on defect morphology and regenerative conditions [12]. This distinction is especially relevant in large apicomarginal or through-and-through defects, where loss of cortical bone may facilitate soft-tissue invasion, impair space maintenance, and compromise predictable osseous regeneration [8,13].

To improve healing outcomes, several regenerative biomaterials have been proposed, including membranes, hydroxyapatite, beta-tricalcium phosphate, xenografts, and autologous platelet concentrates [8,14]. Whereas bone substitutes mainly provide osteoconductive scaffolding, platelet-rich fibrin (PRF) acts as a biologically active fibrin matrix enriched with platelets, leukocytes, cytokines, and growth factors such as PDGF, TGF-β1, VEGF, and IGF-1 [15,16,17]. These bioactive mediators are released gradually and may enhance angiogenesis, fibroblast proliferation, extracellular matrix formation, and osteoblastic differentiation [8,16,18].

PRF is considered a second-generation platelet concentrate prepared without anticoagulants or exogenous activators [18,19]. Different formulations—including conventional PRF, leukocyte-rich PRF (L-PRF), advanced PRF (A-PRF), A-PRF+, and injectable PRF (i-PRF)—differ in centrifugation protocol, fibrin architecture, cellular distribution, and release kinetics [15,16]. Consequently, these preparations should not necessarily be interpreted as biologically equivalent interventions.

In periodontology, implant dentistry, extraction socket preservation, and regenerative endodontics, PRF has demonstrated promising effects on soft-tissue maturation, postoperative discomfort, and early bone formation [15,16,20]. These biological advantages have stimulated interest in its use as an adjunctive material in EMS. Several randomized controlled trials and prospective comparative studies have evaluated PRF placement within the periapical surgical crypt following root-end surgery [13,20,21,22,23,24]. Some studies reported improvements in early bone fill, CBCT volumetric reduction, postoperative pain, edema, or quality of life, whereas others failed to demonstrate statistically significant advantages [23,24,25,26].

Assessment methodology may partly explain these discrepancies. Conventional periapical radiographs provide two-dimensional information and are susceptible to geometric distortion and superimposition. In contrast, cone-beam computed tomography (CBCT) permits three-dimensional volumetric analysis, evaluation of cortical plate regeneration, and more sensitive assessment of healing [27,28]. As CBCT-based outcome measures become increasingly incorporated into endodontic microsurgery studies, a comprehensive synthesis integrating evidence across different imaging modalities and clinically relevant outcomes is warranted.

Although previous systematic reviews have suggested that PRF may reduce postoperative pain, they failed to demonstrate consistent improvements in radiographic healing or CBCT volumetric bone regeneration [29]. Moreover, available evidence has not been comprehensively synthesized according to clinically relevant variables such as defect morphology, PRF formulation, imaging modality, comparator type, and patient-centered outcomes, thereby limiting the clinical applicability of current evidence.

Therefore, the aim of the present systematic review and meta-analysis was to evaluate the efficacy of PRF and its derivatives as adjuncts in endodontic surgery by separately analyzing patient-centered postoperative outcomes and radiographic and volumetric bone-healing outcomes. Specifically, this review aimed to determine whether PRF improves bone regeneration, overall surgical success, postoperative pain and inflammation, soft-tissue healing, and quality of life compared with surgery performed without PRF.

## 2. Materials and Methods

### 2.1. Protocol and Registration

This systematic review and meta-analysis was conducted and reported in accordance with the PRISMA 2020 statement [30] (Supplementary File S1), PRISMA-S recommendations [31], and the Cochrane Handbook for Systematic Reviews of Interventions [32]. The review protocol was registered in PROSPERO prior to study initiation (protocol registration number: CRD420261400163).

### 2.2. Review Question

In patients undergoing endodontic surgery or endodontic microsurgery, does the adjunctive use of platelet-rich fibrin (PRF) and its derivatives (L-PRF, A-PRF, A-PRF+, and i-PRF), compared with surgery performed without PRF, improve bone regeneration, radiographic healing, overall surgical success, soft-tissue healing, postoperative pain and inflammation, or patient quality of life?

### 2.3. Eligibility Criteria

Detailed inclusion and exclusion criteria for each eligibility domain are presented in Table 1. Randomized controlled trials and comparative clinical studies evaluating platelet-rich fibrin (PRF) or its derivatives as adjuncts to EMS for lesions of endodontic origin were included. Primary outcomes were bone regeneration assessed by cone-beam computed tomography (CBCT) and radiographic healing. Secondary outcomes included postoperative pain, edema, quality of life, soft-tissue healing, periodontal parameters, bone density, and overall clinical–radiographic success.

### 2.4. Search Strategy

A comprehensive electronic search was conducted in MEDLINE/PubMed, Embase, Scopus, Web of Science, and Cochrane CENTRAL from database inception to April 2026. Reference lists of all included studies and relevant reviews were also hand-searched to identify additional eligible studies. The electronic searches were independently performed by three reviewers (J.J.S.-E., C.V.-P., and P.M.-M.), who developed the search strategies, conducted the searches across all information sources, and verified the retrieved records prior to study selection. The search strategy combined terms related to platelet-rich fibrin (PRF), endodontic surgery, and bone healing outcomes (Supplementary Table S1). No language restrictions were applied. Titles and abstracts published in languages familiar to the review team were screened directly, whereas records in other languages were assessed using English titles and abstracts when available. If a potentially eligible study had been identified in another language, the full text would have been translated using reliable translation tools and, when necessary, reviewed with the assistance of a native or proficient speaker. However, no eligible studies requiring this procedure were identified.

### 2.5. Selection of Studies

All records retrieved from the electronic databases were exported to reference management software (Mendeley Desktop) to identify and remove duplicates. After deduplication, the remaining records were imported into the Rayyan screening platform (Qatar Computing Research Institute) to facilitate study selection.

Electronic and manual searches identified the studies included in the PRISMA flow diagram. After duplicate removal and full-text assessment for eligibility, seventeen clinical reports corresponding to sixteen independent clinical datasets met the inclusion criteria and were included in the qualitative synthesis.

The study selection process was conducted in two stages: title and abstract screening to exclude clearly irrelevant studies, and full-text review of potentially eligible articles according to the predefined inclusion and exclusion criteria.

Three reviewers (J.J.S.-E., C.V.-P., and P.M.-M.) independently screened titles, abstracts, and full-text articles for eligibility. Disagreements regarding study inclusion were resolved through discussion and consensus.

To avoid double-counting of data, a distinction was made between individual reports and independent clinical datasets. This was particularly relevant for the publications reporting different outcomes derived from the same factorial randomized clinical trial [20,22].

Manual searches of selected journals and reference lists of included studies were also performed.

**Figure 1:**
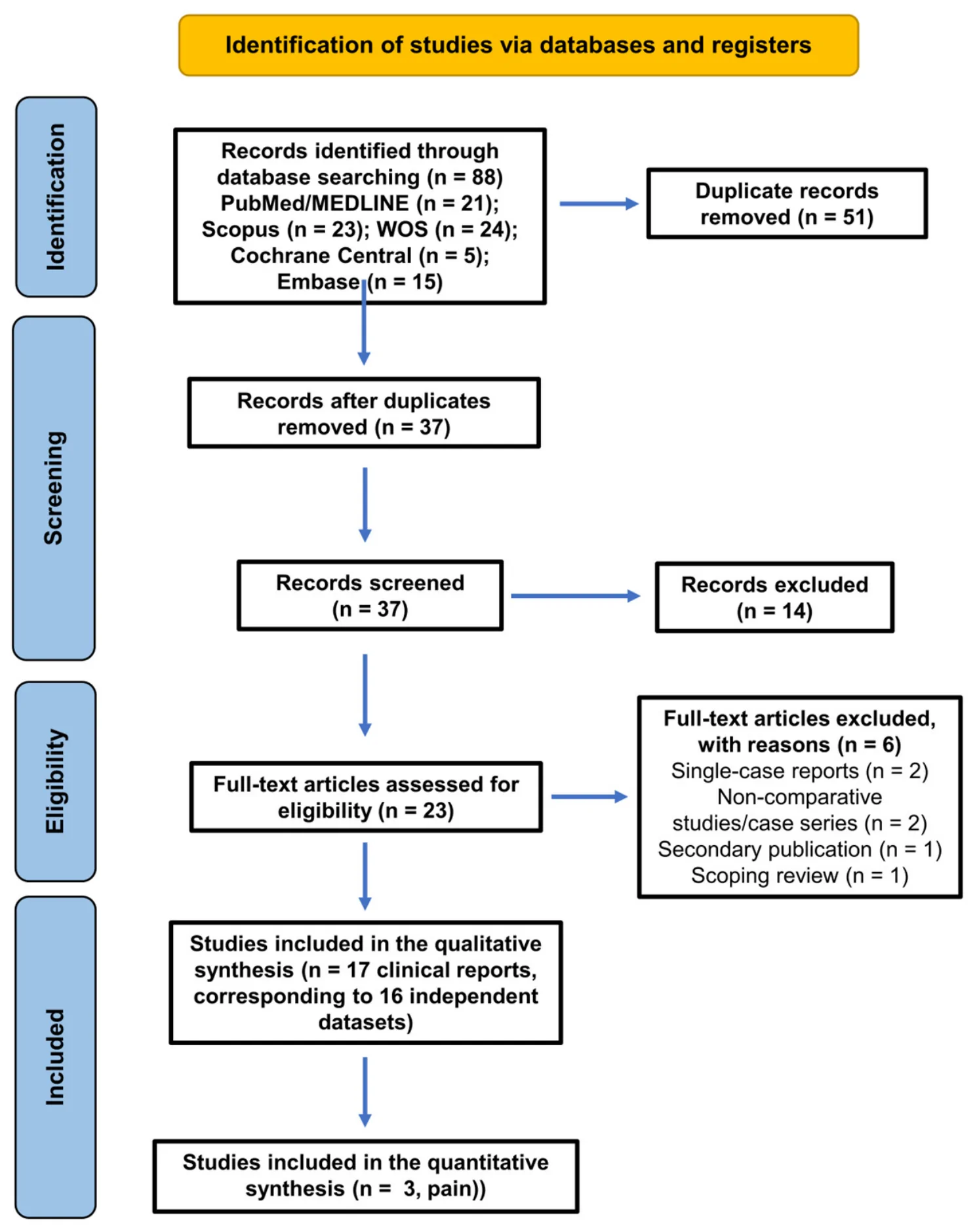
PRISMA 2020 flow diagram illustrating the study selection process for the systematic review and meta-analysis on the use of platelet-rich fibrin (PRF) and its derivatives in endodontic surgery.

The study selection process is summarized in the PRISMA 2020 flow diagram (Figure 1).

### 2.6. Data Extraction

A standardized data extraction form was developed a priori and pilot-tested before formal data collection. Three reviewers (J.J.S.-E., C.V.-P., and P.M.-M.) independently extracted data from all included studies. Extracted information included study design, country, sample size, participant characteristics, type of periapical defect, surgical procedure, PRF formulation, comparator intervention, follow-up duration, imaging modality, outcome measures, and quantitative results relevant to the review objectives.

When multiple publications reported outcomes from the same clinical dataset, data were carefully examined to avoid duplicate inclusion of participants. In such cases, complementary outcome information was extracted from each report while preserving the integrity of the original dataset. Any discrepancies between reviewers during data extraction were resolved through discussion and consensus. When required information was unclear or incompletely reported, the available data were extracted as reported, and assumptions were explicitly documented during the synthesis process. No automated data extraction tools were used.

### 2.7. Data Synthesis and Statistical Analysis

Continuous outcomes were synthesized using mean difference (MD) when outcomes were measured using the same scale, or standardized mean difference (SMD) with Hedges correction when different scales or instruments were used.

Dichotomous outcomes were synthesized using odds ratios (ORs) or risk ratios (RRs) with corresponding 95% confidence intervals (CIs).

Meta-analysis was performed when at least three studies reported sufficiently comparable outcomes. Meta-analyses were performed using OpenMeta[Analyst] software (Brown University, version 10.10) [33], applying a DerSimonian–Laird random-effects model with inverse-variance weighting to account for between-study variability.

Statistical heterogeneity was evaluated using Cochran’s Q test, the I^2^ statistic, τ^2^ estimates, and clinical plausibility [32]. I^2^ values were interpreted as follows: <25% (low), 25–50% (moderate), 50–75% (substantial), and >75% (considerable heterogeneity).

Heterogeneity was interpreted cautiously, particularly when the number of studies available for pooling was small. When quantitative pooling was not considered methodologically appropriate, findings were synthesized narratively.

Predefined subgroup analyses were conducted according to: PRF formulation, imaging modality, defect morphology, follow-up duration, and comparator type.

### 2.8. Risk of Bias Assessment

Randomized controlled trials were assessed using the revised Cochrane Risk of Bias tool (RoB 2) [34]. The following domains were evaluated: bias arising from the randomization process, bias due to deviations from intended interventions, bias due to missing outcome data, bias in measurement of the outcome, and bias in selection of the reported result. Domain-level judgments and an overall risk-of-bias judgment were assigned to each randomized trial according to RoB 2 guidance.

Non-randomized comparative studies were assessed separately using the Risk Of Bias In Non-randomized Studies of Interventions (ROBINS-I) tool [35]. The following domains were evaluated: bias due to confounding, bias in selection of participants into the study, bias in classification of interventions, bias due to deviations from intended interventions, bias due to missing data, bias in measurement of outcomes, and bias in selection of the reported result. Domain-level judgments and an overall risk-of-bias judgment were assigned to each non-randomized study according to ROBINS-I guidance.

### 2.9. Analysis of GRADE Evidence Levels

The certainty of evidence for each outcome was evaluated using the GRADE (Grading of Recommendations, Assessment, Development and Evaluation) approach [36].

Evidence was classified as high, moderate, low, or very low certainty according to the following domains: risk of bias, inconsistency, indirectness, imprecision, and publication bias. Publication bias was planned to be assessed using funnel plots and Egger’s regression test when at least 10 studies were available [37]. GRADE assessments were performed independently by two reviewers, and disagreements were resolved through discussion and consensus.

Summary judgments were presented in Summary of Findings (SoF) tables for the main outcomes. Because several outcomes included non-randomized comparative studies and heterogeneous methodologies, certainty ratings were interpreted cautiously.

## 3. Results

### 3.1. Study Selection

The study selection process is summarized in the PRISMA 2020 flow diagram (Figure 1). A total of 88 records were identified through electronic database searching. After removal of 51 duplicate records, 37 studies remained for title and abstract screening. Of these, 14 records were excluded during the screening phase. Subsequently, 23 full-text articles were assessed for eligibility. Six studies were excluded after full-text evaluation because they did not meet the predefined inclusion criteria. The full-text articles excluded after eligibility assessment, together with the reasons for exclusion, are listed in Supplementary Table S2.

Ultimately, 17 studies were included in the qualitative synthesis. The final qualitative synthesis included seventeen clinical reports corresponding to sixteen independent datasets because Meschi et al. [20] and Meschi et al. [22] reported different outcomes derived from the same factorial randomized clinical trial.

### 3.2. Characteristics of the Included Studies

The characteristics of the included studies are summarized in Table 2. The included investigations demonstrated substantial heterogeneity regarding study design, PRF formulation, comparator type, defect morphology, imaging methodology, and outcome assessment.

A total of 17 clinical reports corresponding to 16 independent clinical datasets were included [13,20,21,22,23,24,25,26,38,39,40,41,42,43,44,45,46]. Most studies were randomized controlled trials, although several prospective comparative clinical studies were also identified. Sample sizes ranged from 10 [42] to 126 [40] participants, and follow-up periods varied between 7 [43] postoperative days and 12 months.

**Table 2:** Characteristics of the included studies evaluating platelet-rich fibrin (PRF) and its derivatives as adjuncts in endodontic surgery.

Study	Design	Sample	Intervention	Comparator	Defect/Population	Follow-Up/Imaging	Main Outcomes
Dhiman et al. 2015 [13]	Double-blind RCT	30 (15/15)	PRF membrane	No GTR	Apicomarginal defects	12 months; RVG	Periodontal parameters; radiographic healing
Monga et al. 2016 [45]	Comparative clinical study	30 (10/10/10)	PRF + MTA	MTA; MTA + HA	Large maxillary anterior lesions	9 months; IOPAR	Lesion size reduction; clinical healing
Thanikasalam et al. 2018 [46]	Prospective comparative study	14 patients (15 sites)	PRF; PRF + NcHA + collagen	No graft	Large/through-and-through lesions	6 months; RVG	Bone healing; lesion area reduction
Meschi 2018/2020 [20,22]	2 × 2 factorial RCT	50 randomized (44 analyzed)	L-PRF ± Bio-Gide	No L-PRF ± Bio-Gide	Persistent apical periodontitis	1 week–12 months; CBCT, PR, US	Pain, QoL, radiographic and CBCT healing
Soto-Peñaloza et al. 2020 [23]	Parallel-arm RCT	50 (25/25)	A-PRF+	Conventional EMS	Chronic apical periodontitis	7 days	Pain, analgesics, QoL
Karan & Aricioğlu 2020 [38]	Four-arm RCT	40 lesions	PRF; MTA + PRF	GP; MTA	Persistent periapical lesions	12 months; CBCT	Lesion volume; bone density
Singh et al. 2020 [40]	Comparative clinical study	126 (42/group)	PRF	HA; CERAMENT	Maxillary anterior lesions	12 months; PR	Clinical and radiographic outcomes
Tiwari et al. 2021 [39]	Comparative clinical study	20 (5/group)	PRF; PRF + HA; PRF + alendronate	No graft	Anterior lesions > 5 mm	12 months; CBCT	Volumetric bone regeneration
Arpitha et al. 2023 [21]	Double-blind RCT	38 randomized (34 analyzed)	i-PRF + collagen	Conventional EMS	Through-and-through defects	12 months; PR & CBCT	PENN, Molven, cortical closure
You 2023 et al. [41]	Three-arm RCT	18	PRF membrane	Control; CGF	Maxillary anterior lesions	3 & 6 months; CBCT	Bone volume reduction
Kavitha et al. 2024 [42]	Comparative study	10 (5/5)	PRF + β-TCP	PRP + β-TCP	Maxillary incisors	6 & 12 months; CBCT	Bone density
Karkle et al. 2025 [25]	RCT	43	A-PRF	Conventional EMS	Periradicular lesions	6 & 12 months; CBCT	Healing, lesion volume, soft tissues
Gulsever et al. 2025 [26]	Four-arm RCT	64	L-PRF	RG/OG	Large periapical lesions	12 months; PR	Pain, PAI, radiographic healing
Major et al. 2025 [43]	Pilot RCT	20 (10/10)	PRF membrane	Conventional EMS	Periapical surgery	7 days; 3D scan	Pain, edema, QoL
Bhardwaj et al. 2025 [44]	Comparative clinical study	60 (20/group)	PRF	Control; HA	Maxillary anterior lesions	9 months; CBCT & PR	Pain, swelling, bone density
Kumar et al. 2026 [24]	RCT	50 patients (133 teeth)	PRF	Calcium phosphosilicate bone putty	Large periapical lesions	12 months; PR & CBCT	2D and 3D bone healing

Abbreviations: A-PRF, advanced platelet-rich fibrin; CBCT, cone-beam computed tomography; CGF, concentrated growth factor; EMS, endodontic microsurgery; GTR, guided tissue regeneration; HA, hydroxyapatite; i-PRF, injectable platelet-rich fibrin; IOPAR, intraoral periapical radiography; L-PRF, leukocyte-rich platelet-rich fibrin; MTA, mineral trioxide aggregate; PAI, periapical index; PENN, Penn 3D criteria; PR, periapical radiography; PRF, platelet-rich fibrin; PRP, platelet-rich plasma; QoL, quality of life; RCT, randomized controlled trial; RVG, radiovisiography; US, ultrasound.

Different PRF formulations were evaluated across studies, including conventional PRF, leukocyte-rich PRF (L-PRF), advanced PRF (A-PRF), A-PRF+, injectable PRF (i-PRF), and combinations of PRF with biomaterials such as hydroxyapatite, nanocrystalline hydroxyapatite, collagen, beta-tricalcium phosphate, and alendronate.

Comparator groups also varied considerably and included surgery without PRF, empty defect/clot healing, hydroxyapatite, concentrated growth factor (CGF), bone putty, CERAMENT, mineral trioxide aggregate (MTA), membranes, and platelet-rich plasma (PRP).

Studies evaluated different categories of periapical defects, including apicomarginal defects [13], through-and-through lesions [21], large periapical lesions [24,39,45], and conventional periapical lesions requiring endodontic surgery [25,26,38,41,43,44]. Most studies focused on anterior maxillary teeth, although lesion size and anatomical complexity varied substantially among investigations.

Radiographic assessment methods included conventional periapical radiography, radiovisiography (RVG), intraoral periapical radiography (IOPAR), and cone-beam computed tomography (CBCT). CBCT-based studies increasingly incorporated three-dimensional volumetric analyses and bone density measurements [24,25,38,41]. Clinical and patient-reported outcomes included postoperative pain, edema, analgesic consumption, quality of life, periodontal parameters, radiographic healing criteria, and overall surgical success [20,22,23,26,43].

### 3.3. Subgroup Structure and Outcome Synthesis

Because of substantial heterogeneity in outcome definitions, imaging methodologies, PRF formulations, and comparator groups, outcomes were categorized into predefined analytical subgroups to facilitate structured synthesis and interpretation. The outcome classification and synthesis strategy are summarized in Table 3.

**Table 3:** Outcome classification and synthesis strategy.

Outcome Category	Included Studies	Outcome Measure(s)	Synthesis Approach
**Postoperative pain**	Meschi et al. [20,22]; Soto-Peñaloza et al. [23]; Major et al. [43]	VAS pain, analgesic consumption	Random-effects meta-analysis (Hedges’ SMD)
**Edema and inflammation**	Major et al. [43]; Karkle et al. [25]; Bhardwaj et al. [44]; Gulsever et al. [26]	Clinical swelling, 3D facial volumetry	Narrative synthesis
**Quality of life**	Meschi et al. [20,22]; Soto-Peñaloza et al. [23]; Major et al. [43]	QoL questionnaires, PROMIS, functional limitation	Narrative synthesis
**Clinical–radiographic success**	Dhiman et al. [13]; Arpitha et al. [21]; Karkle et al. [25]	Molven, PENN, clinical–radiographic success	Random-effects meta-analysis (OR)
**2D radiographic healing**	Monga et al. [45]; Thanikasalam et al. [46]; Singh et al. [40]; Gulsever et al. [26]	Lesion area reduction, PAI, PALA	Narrative synthesis *
**CBCT bone regeneration**	Karan & Aricioğlu [38]; Tiwari et al. [39]; Arpitha et al. [21]; You et al. [41]; Kavitha et al. [42]; Karkle et al. [25]; Kumar et al. [24]	Defect volume reduction, cortical closure	Narrative synthesis
**Bone density**	Karan & Aricioğlu [38]; Kavitha et al. [42]; Bhardwaj et al. [44]	Grayscale values, bone density (HU)	Narrative synthesis
**Complex defects**	Dhiman et al. [13]; Thanikasalam et al. [46]; Arpitha et al. [21]; Tiwari et al. [39]; Gulsever et al. [26]; Kumar et al. [24]	Healing of apicomarginal, through-and-through, and large lesions	Subgroup narrative synthesis

* Quantitative pooling was planned when outcome definitions and reporting methods were sufficiently homogeneous. **Abbreviations:** CBCT, cone-beam computed tomography; HU, Hounsfield units; OR, odds ratio; PAI, periapical index; PALA, periapical lesion area; PENN, Penn 3D criteria; PROMIS, Patient-Reported Outcomes Measurement Information System; QoL, quality of life; SMD, standardized mean difference; VAS, visual analogue scale. Meta-analysis was performed only when at least three studies reported sufficiently homogeneous outcomes; otherwise, findings were synthesized narratively.

### 3.4. Quantitative and Narrative Synthesis of Outcomes

#### 3.4.1. Postoperative Pain

Postoperative pain was the most consistently reported patient-related outcome across the included studies. Pain intensity was generally assessed using visual analogue scale (VAS) scores, although analgesic protocols, assessment intervals, and reporting methods varied substantially among investigations.

Most randomized clinical trials demonstrated favorable postoperative pain trends in PRF-treated groups compared with controls, particularly during the first postoperative week [20,23,43], although statistically significant intergroup differences were not consistently observed across all investigations [44]. The individual effect reported by Major et al. [43] demonstrated a favorable effect for PRF (Hedges g = 0.99; 95% C.I. = 0.09–1.89).

**Figure 2:**
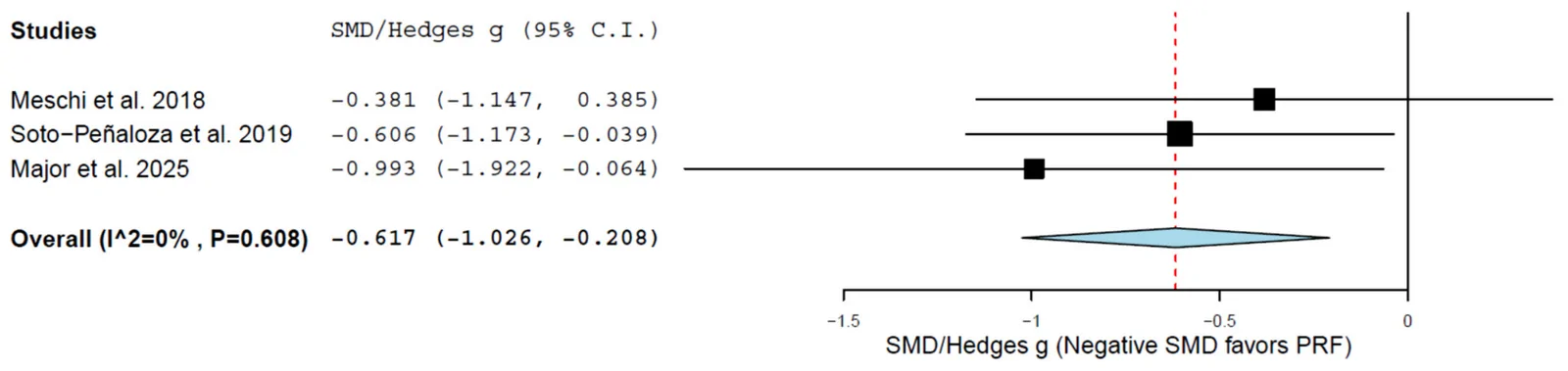
Forest plot of early postoperative pain after endodontic microsurgery comparing PRF-based interventions versus control groups. Random-effects meta-analysis using standardized mean differences (Hedges g). Lower SMD values indicate lower postoperative pain. Negative effect sizes favor PRF for reduction of postoperative pain during the first postoperative week. Squares represent individual study estimates, horizontal lines indicate 95% confidence intervals, and the diamond represents the pooled random-effects estimate (red dash line) [20,23,43].

Postoperative pain was quantitatively synthesized using a random-effects inverse-variance meta-analysis with Hedges’ standardized mean differences (SMDs) and 95% confidence intervals (Figure 2). A random-effects model was prespecified because of the expected clinical heterogeneity in PRF formulations, analgesic regimens, and postoperative pain assessment methods across studies. Three randomized controlled trials evaluating postoperative pain during the first postoperative week were included (Meschi et al. [20,22], Soto-Peñaloza et al. [23], and Major et al. [43]).

The pooled analysis demonstrated a statistically significant reduction in early postoperative pain favoring PRF-based interventions (SMD/Hedges g = −0.617; 95% C.I. = −1.026–−0.208; *p* = 0.003). Heterogeneity was very low (I^2^ = 0%; Tau2 = 0.000; *p* = 0.608). All included studies showed a consistent direction of effect favoring PRF, although effect magnitude varied among investigations. The largest individual effect estimate was observed in Major et al. [43], whereas Meschi et al. [22] demonstrated a smaller and non-significant effect size.

Singh et al. [40] reported shorter time to pain resolution in PRF-treated defects compared with hydroxyapatite and CERAMENT™ groups. However, other studies did not find statistically significant intergroup differences [23,26].

Despite variability in pain scales, follow-up intervals, analgesic regimens, and PRF formulations, the direction of effect was generally consistent across studies. Studies evaluating advanced PRF formulations, including A-PRF+ and L-PRF, also suggested improved postoperative comfort and reduced analgesic consumption compared with surgery performed without PRF [23,25,43]. Despite differences in pain scales and analgesic protocols, the direction of effect remained consistent across studies [20,23,43]. However, interpretation of pooled estimates should be approached cautiously because of methodological heterogeneity, limited sample sizes, and differences in surgical protocols and outcome assessment methods.

#### 3.4.2. Edema and Inflammation

Postoperative edema and inflammatory symptoms were evaluated in a limited number of studies and were generally assessed using clinical swelling scores, facial measurements, patient-reported discomfort, or three-dimensional volumetric analyses [25,26,43,44].

In most cases, studies evaluating PRF derivatives reported a tendency toward reduced postoperative swelling and inflammatory response compared with surgery performed without PRF or with conventional biomaterials. Major et al. [43] observed significantly lower postoperative edema and improved patient-reported recovery in the PRF group during the first postoperative week following EMS. They used standardized three-dimensional facial surface scanning and volumetric analysis, demonstrating a mean volumetric difference of 7.12 cm^3^ favoring PRF (*p* = 0.025).

Similarly, two studies [25,44] reported favorable postoperative healing trends in PRF-treated sites, although statistically significant reductions in swelling were not consistently observed.

However, substantial heterogeneity was observed regarding edema assessment methods, timing of postoperative evaluation, comparator groups, and PRF formulations. Some studies used subjective clinical scoring systems, whereas others incorporated volumetric or imaging-based approaches, limiting the feasibility of quantitative pooling. On the contrary, Gulsever et al. [26] did not find significant differences in swelling in PRF-treated groups compared with controls.

Because of these methodological differences and the limited number of comparable datasets, findings related to edema and inflammation were synthesized narratively.

#### 3.4.3. Quality of Life Outcomes

Quality of life (QoL) and patient-reported outcome measures were evaluated in a limited number of randomized clinical trials, primarily during the early postoperative period following endodontic microsurgery [20,23,43].

Meschi et al. [20,22] primarily evaluated postoperative quality-of-life outcomes, while the 1-year imaging analysis demonstrated no significant improvement in bone healing with L-PRF. Similarly, Soto-Peñaloza et al. [23] observed lower postoperative discomfort, improved functional recovery, and reduced analgesic consumption in the A-PRF+ group compared with controls during the first postoperative week.

Major et al. [43] incorporated PROMIS-based assessments and three-dimensional facial analysis, demonstrating favorable postoperative recovery patterns in patients receiving PRF membranes. Improvements were mainly associated with pain reduction, decreased swelling, and better early functional comfort. They demonstrated significant improvements in physical function, sleep disturbance, pain interference, social participation, and cognitive function in patients treated with PRF membranes.

Considerable heterogeneity existed among studies regarding QoL instruments, follow-up intervals, outcome definitions, and PRF formulations. Some investigations evaluated global quality of life, whereas others focused on functional limitation, postoperative symptoms, or patient satisfaction. Because of this variability and the limited number of studies, quantitative pooling was not considered methodologically appropriate.

#### 3.4.4. Clinical and Radiographic Success

Clinical and radiographic success was evaluated using different healing criteria, including Molven classification, PENN 3D criteria, periapical index (PAI), clinical symptom resolution, and combined clinical–radiographic assessments [13,21,25,26].

Overall, studies evaluating PRF derivatives demonstrated favorable healing trends compared with control groups, particularly in complex defects and lesions with extensive bone destruction. Dhiman et al. [13] reported improved periodontal healing and clinical success in apicomarginal defects treated with PRF membranes, but did not find significant differences in overall clinical–radiographic healing success between PRF and control groups. Similarly, Arpitha et al. [21] observed enhanced radiographic healing, reduction in defect size, and improved cortical closure in through-and-through lesions managed with i-PRF and collagen membranes.

Radiographic healing outcomes were inconsistent across studies. Karkle et al. [25] evaluated radiographic healing using Molven and modified PENN 3D criteria and observed progressive healing in both groups during 6- and 12-month follow-up evaluations, without demonstrating consistent superiority of A-PRF over conventional microsurgery. Gulsever et al. [26] reported favorable radiographic healing trends in groups receiving adjunctive L-PRF, although no statistically significant intergroup differences were observed in PAI scores. Meschi et al. [20,22] found no significant improvement in PRF-treated groups using CBCT or ultrasound assessment. Similarly, Monga et al. [45] did not observe statistically significant differences between PRF and hydroxyapatite-treated defects.

Despite the generally favorable direction of effect, important heterogeneity existed among studies regarding healing criteria, imaging modalities, lesion morphology, follow-up duration, and comparator groups. Some investigations relied exclusively on two-dimensional radiographic interpretation, whereas others incorporated CBCT-based volumetric analyses and three-dimensional healing criteria.

**Figure 3:**
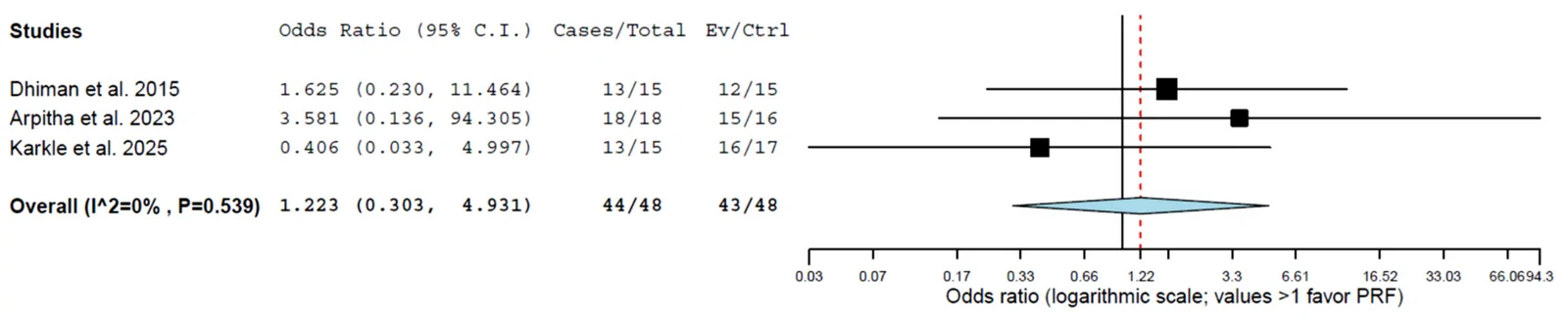
Forest plot of clinical–radiographic success following endodontic microsurgery comparing PRF-based interventions versus control groups. Random-effects meta-analysis using odds ratios (OR). Values greater than 1 favor PRF. Random-effects estimates include continuity correction for zero-event cells. Factorial non-decomposable studies were not included in the pooled analysis. Squares represent individual study estimates, horizontal lines indicate 95% confidence intervals, and the diamond represents the pooled random-effects estimate (red dash line) [13,21,25].

Quantitative synthesis of dichotomous healing outcomes was performed using a random-effects meta-analysis with odds ratios (ORs). Three randomized controlled trials evaluating clinical–radiographic healing after endodontic microsurgery were included [13,21,25] (Figure 3).

The pooled analysis demonstrated no statistically significant difference between PRF-based interventions and control groups regarding overall clinical–radiographic success (OR = 1.22 [95% CI: 0.30–4.93]; I^2^ = 0%; *p* = 0.539). Although Dhiman et al. [13] and Arpitha et al. [21] numerically favored PRF-based interventions, Karkle et al. [25] showed a direction favoring the control group. Overall, the pooled effect estimate was imprecise and associated with wide confidence intervals, reflecting the limited number of available studies and the high frequency of successful healing in both intervention and control groups.

#### 3.4.5. Two-Dimensional Radiographic Healing

Two-dimensional radiographic healing was evaluated using conventional periapical radiography, radiovisiography (RVG), intraoral periapical radiography (IOPAR), periapical index (PAI) scores, and periapical lesion area (PALA) measurements [26,38,45]. Several studies assessed changes in lesion size or radiographic density over follow-up periods ranging from 6 to 12 months.

Monga et al. [45] reported greater reduction in radiolucency size in the PRF group compared with MTA alone, whereas differences between PRF and hydroxyapatite groups were not statistically significant. Thanikasalam et al. [46] also reported the greatest radiographic healing in defects treated with PRF combined with nanocrystalline hydroxyapatite and collagen, whereas PRF alone demonstrated less complete healing.

Similarly, Gulsever et al. [26] demonstrated improved PAI and PALA outcomes in groups receiving L-PRF adjunctively during surgical treatment. Other studies evaluating conventional radiographic healing trends reported favorable reductions in radiolucency and improved healing dynamics in PRF-treated defects, particularly regarding periapical lesion area measurements [38,40]. Singh et al. [40] reported smaller residual radiographic lesion areas after surgical intervention in PRF- and CERAMENT™-treated defects compared with hydroxyapatite-treated defects.

However, substantial heterogeneity existed among studies regarding radiographic criteria, imaging calibration, lesion morphology, and methods used to quantify healing. Two-dimensional radiographic interpretation also presents inherent limitations because of anatomical superimposition and reduced sensitivity for detecting volumetric bone changes.

Because of variability in outcome reporting and measurement methodology, quantitative pooling was considered feasible only in selected comparisons, whereas several outcomes were synthesized narratively.

#### 3.4.6. CBCT Volumetric Bone Regeneration

Cone-beam computed tomography (CBCT)-based volumetric assessment was increasingly incorporated in recent studies to evaluate three-dimensional bone regeneration following endodontic microsurgery [21,24,25,38,39,41,42]. Compared with conventional two-dimensional radiography, CBCT allowed more detailed assessment of lesion volume reduction, cortical bone repair, and bone density changes over time.

Karan & Aricioğlu [38] found significantly improved volumetric healing in the MTA and MTA + PRF groups compared with controls, whereas PRF alone did not significantly differ from the control group. Similarly, Tiwari et al. [39] reported the greatest volumetric defect reduction in defects treated with PRF combined with hydroxyapatite, whereas PRF alone and PRF combined with alendronate demonstrated outcomes comparable to controls.

You et al. [41] found that PRF and CGF demonstrated significantly greater early volumetric bone healing than controls at 3 months, although intergroup differences were no longer significant at 6 months. Arpitha et al. [21] reported substantial cortical window reduction and three-dimensional healing in through-and-through lesions in both groups, without statistically significant intergroup differences associated with i-PRF.

Kavitha et al. [42] evaluated postoperative bone healing using CBCT-derived densitometric analysis and observed progressive increases in bone density values in both PRF + β-TCP and PRP + β-TCP groups during follow-up.

More recent randomized clinical trials incorporated standardized CBCT volumetric analyses to evaluate healing outcomes associated with advanced PRF formulations. Karkle et al. [25] observed substantial volumetric reduction in both groups over time; however, A-PRF did not demonstrate reproducible superiority over conventional microsurgery regarding CBCT healing outcomes. Kumar et al. [24] reported significantly greater 2D area reduction with calcium phosphosilicate-based bone putty compared with PRF in large periapical lesions. Despite generally favorable outcomes, substantial heterogeneity existed regarding CBCT acquisition protocols, segmentation methods, volumetric software, follow-up duration, and defect morphology. Some studies reported absolute volumetric reduction, whereas others expressed percentage reduction or combined volumetric and density outcomes, limiting direct quantitative comparability. Meschi et al. [22] found no significant improvement in volumetric CBCT healing with L-PRF, whereas Bio-Gide^®^ membrane use was associated with superior healing outcomes.

Because of methodological variability and differences in reporting standards, synthesis of CBCT outcomes was primarily descriptive and stratified according to lesion type and PRF formulation.

#### 3.4.7. Bone Density Outcomes

Bone density outcomes were evaluated in a limited number of studies using CBCT-derived grayscale measurements or Hounsfield unit (HU)-related assessments to estimate mineralized tissue regeneration within the surgical defect [38,42,44].

Karan & Aricioğlu [38] observed postoperative increases in grayscale density values in all groups, without significant intergroup differences in postoperative density measurements. Similarly, Bhardwaj et al. [44] observed higher CBCT-derived bone density values and healing trends in lesions treated with PRF compared with hydroxyapatite and conventional control groups. Kavitha et al. [42] compared PRF combined with beta-tricalcium phosphate (β-TCP) versus platelet-rich plasma (PRP) combined with β-TCP and reported significant increases in CBCT-derived bone density values over time in both PRF + β-TCP and PRP + β-TCP groups, without statistically significant intergroup differences. Despite these generally positive findings, important methodological limitations were identified. Studies used different imaging systems, grayscale calibration methods, and density measurement protocols, reducing comparability across investigations. Furthermore, CBCT grayscale values do not represent true Hounsfield units in the same manner as conventional computed tomography, which may limit standardization and interpretation of density measurements.

Because of heterogeneity in imaging methodology, reporting standards, and outcome definitions, quantitative pooling was not considered methodologically appropriate.

#### 3.4.8. Complex Defect Subgroup Analysis

A dedicated subgroup analysis was performed for complex periapical defects, including apicomarginal lesions, through-and-through defects, and large periapical lesions characterized by extensive cortical bone destruction [13,21,24,26,39,45,46].

Dhiman et al. [13] reported high healing rates in apicomarginal defects irrespective of PRF use, with no significant superiority of PRF over controls. Arpitha et al. [21] investigated through-and-through lesions using i-PRF combined with collagen membranes and reported favorable healing outcomes and cortical window reduction in both groups, without significant intergroup differences in PENN healing classification or volumetric defect reduction.

Studies focusing on large periapical lesions also reported favorable regenerative trends associated with PRF-based approaches. Monga et al. [45] and Thanikasalam et al. [46] reported complete radiographic healing in through-and-through and large lesions treated with PRF combined with nanocrystalline hydroxyapatite and collagen. Similarly, Kumar et al. [24] demonstrated enhanced three-dimensional bone regeneration in extensive lesions managed with PRF compared with bone putty.

Gulsever et al. [26] further suggested that adjunctive L-PRF may improve radiographic and clinical healing in surgically challenging lesions requiring regenerative management. Overall, studies involving complex defects suggested potential regenerative advantages associated with PRF derivatives; however, findings remained heterogeneous and were not consistently associated with superior outcomes across all radiographic and clinical parameters.

Nevertheless, substantial heterogeneity was present regarding lesion morphology, defect classification, regenerative protocols, imaging methodology, and outcome assessment. The limited number of studies and variability in comparator groups also restricted the feasibility of formal quantitative subgroup pooling.

In defects requiring space maintenance, including large lesions, through-and-through defects, and apicomarginal defects, PRF alone did not consistently demonstrate reproducible superiority over biomaterials providing structural support [13,21,24,39]. Kumar et al. [24] reported significantly greater 3D volumetric bone regeneration with calcium phosphosilicate-based bone putty compared with PRF in large periapical lesions.

### 3.5. Risk of Bias Assessment

Risk-of-bias assessments were conducted separately according to study design. Randomized controlled trials were evaluated using RoB 2, with domain-level and overall judgments presented in Table 4A. All randomized trials were judged as having some concerns, with the most frequent concerns relating to the randomization process, deviations from intended interventions, outcome measurement, and selection of the reported result.

Non-randomized comparative studies were evaluated separately using ROBINS-I, with domain-level and overall judgments presented in Table 4B. All six non-randomized studies were judged to have a serious overall risk of bias, primarily because of susceptibility to confounding. Additional concerns were identified regarding participant selection, deviations from intended interventions, outcome measurement, and selection of the reported result.

Overall, non-randomized comparative studies showed greater methodological uncertainty than randomized controlled trials, particularly because of their susceptibility to confounding and selection bias.

Because of the heterogeneity in study design, PRF protocols, comparator groups, and outcome definitions, the overall certainty and interpretation of pooled estimates were considered cautiously during evidence synthesis. Nevertheless, several methodological limitations remained common across studies, including small sample sizes, inability to blind operators, incomplete outcome reporting, attrition during follow-up, and heterogeneity in units of analysis.

### 3.6. Certainty of Evidence (GRADE)

The certainty of evidence assessed using the GRADE framework was generally low to very low across most evaluated outcomes because of methodological limitations, small sample sizes, imprecision, and substantial clinical heterogeneity among studies (Table 5).

The most consistent evidence was observed for early postoperative pain reduction, where PRF-based interventions demonstrated a favorable direction of effect across studies; however, certainty was downgraded because of the limited number of available studies. Outcomes related to edema, inflammation, and quality of life also suggested potentially favorable postoperative effects associated with PRF, although the certainty of evidence remained low because of heterogeneous outcome measures and limited replication across studies.

Clinical–radiographic healing success and CBCT volumetric outcomes demonstrated very low certainty of evidence due to wide confidence intervals, sparse event distributions, heterogeneous healing criteria, variability in imaging methodologies, and inconsistent directions of effect. Similarly, evidence regarding bone density outcomes and comparisons between PRF and active biomaterials remained very uncertain because of heterogeneous biomaterials, indirect comparisons, and inconsistent quantitative findings. Overall, current evidence suggests that PRF and its derivatives may improve selected postoperative outcomes after endodontic surgery, although robust superiority over conventional biomaterials or control interventions has not yet been conclusively demonstrated.

## 4. Discussion

The present systematic review suggests that the clinical effects of PRF and its derivatives in endodontic surgery are strongly outcome-dependent. The most consistent evidence indicates potential benefits during the early postoperative period, particularly regarding pain reduction and patient recovery, whereas evidence related to long-term bone regeneration and radiographic healing remains heterogeneous and inconclusive.

### 4.1. Postoperative Recovery Outcomes

Postoperative pain was the outcome demonstrating the greatest quantitative consistency across studies. The findings of Major et al. [43] are consistent with the overall evidence indicating reduced early postoperative pain following PRF use. The present meta-analysis further demonstrated a significant reduction in early postoperative pain favoring PRF-based interventions. Despite differences in pain scales, analgesic regimens, and postoperative assessment protocols, the direction of effect remained consistent across studies. However, these quantitative estimates should be interpreted cautiously because of the limited number of directly combinable studies, methodological heterogeneity, variability in PRF formulations and preparation protocols, differences in surgical protocols, and outcome assessment methods. Despite the low statistical heterogeneity observed in the pooled analysis, the consistency in effect direction across all included trials suggests that PRF and its derivatives may contribute to improved early postoperative recovery following endodontic microsurgery.

Evidence regarding edema and quality of life also tends to favor PRF-based interventions, particularly in studies incorporating patient-reported outcome measures or three-dimensional facial analysis techniques [23,43,47,48]. However, substantial heterogeneity in outcome definitions, PROM instruments, and postoperative assessment methodologies limits direct comparability across studies. Future clinical trials should therefore incorporate validated PROMs, standardized analgesic protocols, and objective volumetric swelling assessment methods. Nevertheless, the overall direction of evidence suggested that PRF may contribute to improved modulation of the early postoperative inflammatory response following endodontic microsurgery.

Overall, the available evidence suggested that PRF derivatives may contribute to improved patient-centered postoperative outcomes and early recovery following endodontic surgery. However, the certainty of evidence remained limited because of small sample sizes, heterogeneity in outcome assessment, and risk of bias concerns.

### 4.2. PRF and Bone Regeneration Outcomes

In contrast, current evidence does not support a robust or reproducible effect of PRF on long-term osseous regeneration. CBCT-based studies suggest that PRF may accelerate early healing in certain clinical contexts, although this apparent advantage tends to diminish during longer follow-up periods [24,25,38,39,41]. This finding is biologically plausible because PRF primarily acts through the early release of cytokines and growth factors involved in wound healing, whereas regeneration of extensive periapical defects also depends on structural stability, maintenance of regenerative space, and long-term scaffold support.

Nevertheless, the available evidence suggested that PRF derivatives may contribute to volumetric bone regeneration and three-dimensional healing following endodontic microsurgery, although findings were heterogeneous and not consistently superior to control or active biomaterial groups.

These findings suggest that PRF and its derivatives may not provide a consistent or reproducible improvement in long-term clinical–radiographic healing outcomes following endodontic microsurgery. However, interpretation should remain cautious because of differences in defect morphology, healing criteria, regenerative protocols, and imaging methodologies among studies.

Overall, the available evidence suggested that PRF derivatives may improve two-dimensional radiographic healing following endodontic microsurgery, although interpretation should be approached cautiously because of methodological heterogeneity and imaging limitations.

However, available evidence suggests that PRF derivatives may contribute to improved mineralized tissue formation and radiographic bone density during postoperative healing following endodontic microsurgery, although current evidence remains limited and heterogeneous.

An additional aspect that deserves consideration is the distinction between platelet-rich fibrin (PRF) and plasma rich in growth factors (PRGF) in endodontic surgery. Although both are autologous platelet-derived preparations intended to enhance wound healing and tissue regeneration, important biological and methodological differences exist between them [49]. PRGF is generally obtained through plasma fractionation protocols that exclude leukocytes and produce a liquid or gel-like concentrate with relatively controlled growth factor release kinetics [50], whereas PRF forms a fibrin-rich matrix containing platelets, leukocytes, and cytokines that are gradually released during fibrin degradation [16,17].

From a biological perspective, PRF may provide a more prolonged release of growth factors and improved scaffold stability because of its dense fibrin architecture, whereas PRGF has been associated with greater standardization and reduced inflammatory cell content [49]. These differences may influence postoperative inflammatory modulation, angiogenesis, and early tissue maturation. Clinical experience in other surgical fields has shown that autologous PRGF-based treatments can be safely delivered and may improve patient-centered outcomes such as pain, although their clinical efficacy may remain modest and highly dependent on the treated tissue and application protocol [51].

Nevertheless, direct comparisons between PRF and PRGF in endodontic microsurgery remain extremely limited, and currently available studies do not allow robust conclusions regarding superiority of one platelet concentrate over another.

However, both platelet concentrates share common methodological limitations in the available literature, including heterogeneous preparation protocols, variability in centrifugation parameters, inconsistent outcome measures, and relatively small sample sizes.

Therefore, current evidence does not support interpreting PRF and PRGF as biologically equivalent or interchangeable interventions. Future comparative randomized clinical trials directly evaluating PRF versus PRGF in standardized endodontic microsurgery protocols would be valuable to clarify potential differences in postoperative recovery, volumetric bone healing, and long-term regenerative outcomes.

### 4.3. PRF Versus Biomaterials in Complex Defects

This distinction is particularly relevant in large, non-contained, through-and-through, or apicomarginal lesions. In such situations, PRF alone should not be interpreted as a substitute for barrier membranes or osteoconductive graft materials requiring structural support and maintenance of regenerative space [13,21,24,40,42,45,46]. Importantly, studies comparing PRF with active biomaterials address a substantially different clinical question from studies comparing PRF with surgery alone. The more favorable long-term volumetric outcomes reported for certain grafting materials further support the interpretation that PRF may function more effectively as a biological adjunct or enhancer of wound healing rather than as a stand-alone regenerative scaffold.

Despite these limitations, the available evidence suggested that PRF derivatives may provide particular clinical benefit in complex periapical defects where space maintenance, angiogenesis, and soft-tissue exclusion are critical for successful bone regeneration following endodontic microsurgery. These findings suggest that PRF may function more effectively as a biological adjunct promoting angiogenesis and soft-tissue healing rather than as a substitute for osteoconductive graft materials in structurally compromised defects.

### 4.4. Methodological Considerations and Certainty of Evidence

Another important finding of the present review was the substantial heterogeneity among included studies. Considerable variability existed in PRF preparation protocols, centrifugation parameters, defect morphology, imaging modalities, healing criteria, outcome definitions, and follow-up intervals. This methodological and clinical heterogeneity limited the feasibility of global quantitative synthesis and suggests the need for outcome-specific and subgroup-based interpretation of the available evidence.

Methodological limitations were frequent across the included studies. Although all randomized clinical trials were judged as having some concerns, several studies presented limitations related to the reporting of randomization methods, allocation concealment, or blinding of participants and outcome assessors. In addition, some studies reported incomplete follow-up data or limited information regarding attrition. Non-randomized comparative studies showed greater methodological uncertainty, particularly because of potential confounding and selection bias, as reflected in the ROBINS-I assessment. Small sample sizes and substantial variability in outcome assessment methods and imaging protocols further increased imprecision and reduced comparability across studies. Different PRF formulations and combinations with biomaterials were also analyzed together because the available evidence was insufficient to permit robust formulation-specific meta-analyses. Consequently, the certainty of evidence should be interpreted separately for each specific outcome rather than globally for the review as a whole.

Importantly, GRADE assessments indicated low to very low certainty for most regenerative outcomes, suggesting that current conclusions regarding long-term bone healing should be interpreted cautiously. In contrast, evidence supporting improvements in early postoperative recovery demonstrated greater consistency across studies, although certainty remained limited by sample size and methodological heterogeneity.

### 4.5. Clinical Implications and Future Directions

From a clinical perspective, current evidence suggests that PRF and its derivatives may be reasonably used to improve postoperative comfort and support early soft-tissue healing after endodontic microsurgery (EMS). However, clinicians should avoid interpreting PRF as a predictable substitute for barrier membranes or osteoconductive graft materials in defects requiring structural support and space maintenance. Treatment planning should therefore be individualized according to the primary therapeutic objective, including postoperative recovery, soft-tissue healing, radiographic repair, or long-term volumetric bone regeneration.

Future research should prioritize multicenter randomized controlled trials with adequately powered sample sizes, standardized PRF preparation protocols, CBCT-based volumetric assessment methods, validated patient-reported outcome measures (PROMs), and subgroup analyses according to defect morphology and comparator type.

## 5. Conclusions

Within the limitations of the available evidence, platelet-rich fibrin (PRF) may be a useful adjunct in endodontic microsurgery for improving early postoperative recovery, particularly by reducing postoperative pain during the first postoperative week. However, current evidence does not demonstrate a consistent benefit of PRF for long-term bone regeneration or overall clinical–radiographic healing compared with conventional microsurgery. Because the certainty of evidence ranges from moderate to very low and the available studies are heterogeneous, further well-designed randomized controlled trials using standardized CBCT-based outcome measures are required to clarify the role of PRF in endodontic microsurgery.

## Figures and Tables

**Table 1:** Eligibility criteria used for study selection.

Domain	Inclusion Criteria	Exclusion Criteria
**Context**	Regenerative adjunctive procedures performed during endodontic microsurgery or periapical surgery for periapical lesions of endodontic origin.	Procedures unrelated to endodontic surgery.
**Types of studies**	Randomized controlled trials (RCTs), prospective comparative clinical studies, and parallel-group or split-mouth clinical designs.	Case reports, uncontrolled case series, retrospective studies without a comparator, animal or in vitro studies, narrative reviews, editorials, conference abstracts, protocols without results.
**Population**	Patients of any age undergoing endodontic microsurgery, periapical surgery, apical surgery, root-end surgery, or periradicular surgery for periapical lesions of endodontic origin.	Studies involving implant surgery, periodontal regenerative surgery unrelated to endodontic lesions, intentional replantation, tooth extraction procedures, regenerative endodontic procedures in immature teeth, or non-human subjects.
**Intervention**	Platelet-rich fibrin (PRF) or its derivatives, including PRF, L-PRF, A-PRF, A-PRF+, i-PRF, alone or combined with biomaterials.	Studies evaluating regenerative procedures without PRF derivatives, isolated bone graft materials, pharmacological agents, or non-autologous platelet concentrates.
**Comparators**	Surgery performed without PRF, spontaneous clot healing, hydroxyapatite, MTA, membranes, platelet-rich plasma (PRP), concentrated growth factor (CGF), CERAMENT, bone putty, or other active biomaterials.	Studies without an appropriate comparator group or exclusively comparing different PRF formulations.
**Outcomes**	Primary outcomes: bone regeneration assessed by CBCT volumetric analysis and radiographic healing (PAI, Molven, PENN, or equivalent criteria). Secondary outcomes: postoperative pain, analgesic consumption, edema/swelling, quality of life, soft-tissue healing, periodontal parameters, bone density, and overall clinical–radiographic success.	Studies not reporting at least one relevant clinical or radiographic outcome.

Abbreviations: A-PRF, advanced platelet-rich fibrin; A-PRF+, advanced platelet-rich fibrin plus; CBCT, cone-beam computed tomography; CGF, concentrated growth factor; EMS, endodontic microsurgery; L-PRF, leukocyte-rich platelet-rich fibrin; MTA, mineral trioxide aggregate; PAI, periapical index; PENN, Penn 3D criteria; PRF, platelet-rich fibrin; PRP, platelet-rich plasma. Because terminology varied across the included studies, the terms endodontic microsurgery, periapical surgery, apical surgery, and root-end surgery were accepted according to the nomenclature used by the original authors and were considered clinically equivalent for eligibility purposes.

**Table 4:** (**A**) Risk of bias assessment of randomized controlled trials using the revised Cochrane Risk of Bias tool (RoB 2). (**B**) Risk of bias assessment of non-randomized comparative studies using the Risk Of Bias In Non-randomized Studies of Interventions (ROBINS-I) tool.

(**A**)								
**Study**	**D1**	**D2**	**D3**	**D4**	**D5**			**Overall** **Judgment**
Dhiman 2015 [13]	Low	Some concerns	Low	Low	Some concerns			Some concerns
Meschi 2018/2020 [20,22]	Low	Some concerns	Some concerns	Some concerns	Low			Some concerns
Soto-Peñaloza 2020 [23]	Low	Some concerns	Low	Low	Low			Some concerns
Karan & Aricioğlu 2020 [38]	Some concerns	Some concerns	Some concerns	Some concerns	Some concerns			Some concerns
Arpitha 2023 [21]	Low	Some concerns	Some concerns	Low	Some concerns			Some concerns
You 2023 [41]	Low	Some concerns	Some concerns	Low	Some concerns			Some concerns
Karkle 2025 [25]	Low	Some concerns	Low	Low	Some concerns			Some concerns
Gulsever 2025 [26]	Low	Some concerns	Low	Some concerns	Some concerns			Some concerns
Major 2025 [43]	Low	Some concerns	Low	Low	Some concerns			Some concerns
Kumar 2026 [24]	Low	Some concerns	Low	Low	Some concerns			Some concerns
(**B**)								
**Study**	**D1**	**D2**	**D3**	**D4**	**D5**	**D6**	**D7**	**Overall** **Judgment**
Monga 2016 [45]	Serious	Moderate	Low	Moderate	Low	Moderate	Moderate	Serious
Thanikasalam 2018 [46]	Serious	Moderate	Low	Moderate	Low	Moderate	Moderate	Serious
Singh 2020 [40]	Serious	Moderate	Low	Moderate	Low	Moderate	Moderate	Serious
Tiwari 2021 [39]	Serious	Moderate	Low	Moderate	Low	Moderate	Moderate	Serious
Kavitha 2024 [42]	Serious	Moderate	Low	Moderate	Low	Moderate	Moderate	Serious
Bhardwaj 2025 [44]	Serious	Moderate	Low	Moderate	Moderate	Moderate	Moderate	Serious

Abbreviations: (A) D1, bias arising from the randomization process; D2, bias due to deviations from intended interventions; D3, bias due to missing outcome data; D4, bias in measurement of the outcome; D5, bias in selection of the reported result. Risk-of-bias judgments were performed according to the revised Cochrane Risk of Bias tool for randomized trials (RoB 2). Overall risk-of-bias judgments were derived according to the RoB 2 algorithm. (B) D1, bias due to confounding; D2, bias in selection of participants into the study; D3, bias in classification of interventions; D4, bias due to deviations from intended interventions; D5, bias due to missing data; D6, bias in measurement of outcomes; D7, bias in selection of the reported result. Risk-of-bias judgments were performed according to the Risk Of Bias In Non-randomized Studies of Interventions (ROBINS-I) tool. Overall risk-of-bias judgments were derived according to ROBINS-I guidance.

**Table 5:** GRADE assessment of the certainty of evidence for postoperative and healing outcomes associated with platelet-rich fibrin (PRF) and its derivatives in endodontic surgery.

Outcome	Comparison	Evidence	Main Effect	GRADE Certainty	Justification
Early postoperative pain	PRF/derivatives vs. no PRF	3 reports; ~120 participants	SMD −0.62 [−1.03; −0.21] favoring PRF	Moderate	Downgraded for serious imprecision due to the limited number of studies and participants, despite a consistent direction of effect across the included trials.
Edema/inflammation	PRF vs. control	1 study with 3D measurement + narrative evidence	Reduced edema with PRF in Major 2025	Low	Downgraded for imprecision, single-study evidence, and lack of replication across trials.
Quality of life	PRF/derivatives vs. no PRF	3 reports	Favorable trends in selected functional domains; no pooled estimate	Low	Downgraded for inconsistency, heterogeneous PROM instruments, and absence of pooled quantitative analysis.
Clinical–radiographic success	PRF/derivatives vs. control	3 directly combinable RCTs	OR 1.22 [0.30; 4.93]; not significant	Very low	Downgraded for serious imprecision, sparse event distribution, heterogeneous healing criteria, and high risk of bias across included studies.
CBCT volumetric reduction	PRF vs. control/active biomaterial	Multiple studies; no global pooling	Possible early benefit; inconsistent long-term effect	Very low	Downgraded for substantial heterogeneity in comparators, follow-up periods, volumetric methods, and inconsistency of effect direction.
Bone density	PRF vs. control/HA/PRP + β-TCP	3 studies	General increase in density values without clear PRF superiority	Very low	Downgraded for serious inconsistency, heterogeneous density metrics (HU/GSV), small sample sizes, and indirect comparisons.
PRF vs. active biomaterials	PRF vs. HA/CERAMENT/bone putty	4 studies	Discordant findings; bone putty superior in Kumar 2026 [24]	Very low	Downgraded for major clinical heterogeneity in biomaterials, outcome definitions, and inconsistent comparative effects across studies.

## Data Availability

No new data were created or analyzed in this study. Data sharing is not applicable to this article.

## Supplementary Materials

The following supporting information can be downloaded at: https://www.mdpi.com/article/10.3390/jcm15166392/s1, File S1: PRISMA 2020 Checklist. File S2—Table S1. Literature search in databases. Table S2: Studies excluded from the systematic review and the reasons for exclusion [52,53,54,55,56,57].
