# Supplementary material for: Clinical Outcomes of Platelet-Rich Fibrin in Endodontic Microsurgery: A Systematic Review and Meta-Analysis
Source: J Clin Med. 2026 Aug 18;15(16):6392. doi: 10.3390/jcm15166392 (PMC13513751; doi:10.3390/jcm15166392)
Supplement: Supplementary file 1 [file jcm-15-06392-s001.zip › File S2.pdf]

Supplementary Table S1. Literature search in databases.

Database	Exact Search String Used	No. of Articles	Date of Last Search
PubMed/ MEDLINE	(("platelet-rich fibrin"[Title/Abstract] OR "platelet rich fibrin"[Title/Abstract] OR PRF[Title/Abstract] OR L-PRF[Title/Abstract] OR A-PRF[Title/Abstract] OR "A-PRF+"[Title/Abstract] OR i-PRF[Title/Abstract] OR "autologous platelet concentrate"[Title/Abstract]) AND ("periapical surgery"[Title/Abstract] OR "apical surgery"[Title/Abstract] OR "endodontic surgery"[Title/Abstract] OR "endodontic microsurgery"[Title/Abstract] OR apicoectomy[Title/Abstract] OR "root-end surgery"[Title/Abstract] OR "periradicular surgery"[Title/Abstract]) AND ("periapical lesion"[Title/Abstract] OR "apical periodontitis"[Title/Abstract] OR "bone healing"[Title/Abstract] OR "bone regeneration"[Title/Abstract] OR CBCT[Title/Abstract] OR "through-and-through defect"[Title/Abstract] OR "apicomarginal defect"[Title/Abstract]))	21	28 April 2026
Scopus	TITLE-ABS-KEY(("platelet-rich fibrin" OR "platelet rich fibrin" OR PRF OR L-PRF OR A-PRF OR "A-PRF+" OR i-PRF OR "autologous platelet concentrate") AND ("periapical surgery" OR "apical surgery" OR "endodontic surgery" OR "endodontic microsurgery" OR apicoectomy OR "root-end surgery" OR "periradicular surgery") AND ("periapical lesion" OR "apical periodontitis" OR "bone healing" OR "bone regeneration" OR CBCT OR "through-and-through defect" OR "apicomarginal defect"))	23	28 April 2026
Web of Science	TS=(("platelet-rich fibrin" OR "platelet rich fibrin" OR PRF OR L-PRF OR A-PRF OR "A-PRF+" OR i-PRF OR "autologous platelet concentrate") AND ("periapical surgery" OR "apical surgery" OR "endodontic surgery" OR "endodontic microsurgery" OR apicoectomy OR "root-end surgery" OR "periradicular surgery") AND ("periapical lesion" OR "apical periodontitis" OR "bone healing" OR "bone regeneration" OR CBCT OR "through-and-through defect" OR "apicomarginal defect"))	24	28 April 2026
Cochrane Central	("platelet-rich fibrin" OR "platelet rich fibrin" OR PRF OR L-PRF OR A-PRF OR "A-PRF+" OR i-PRF OR "autologous platelet concentrate") AND ("periapical surgery" OR "apical surgery" OR "endodontic surgery" OR "endodontic microsurgery" OR apicoectomy OR "root-end surgery" OR "periradicular surgery") AND ("periapical lesion" OR "apical periodontitis" OR "bone healing" OR "bone regeneration" OR CBCT OR "through-and-through defect" OR "apicomarginal defect")	5	28 April 2026
EMBASE	('platelet rich fibrin':ti,ab,kw OR 'platelet-rich fibrin':ti,ab,kw OR PRF:ti,ab,kw OR L-PRF:ti,ab,kw OR A-PRF:ti,ab,kw OR 'A-PRF+':ti,ab,kw OR i-PRF:ti,ab,kw OR 'autologous platelet concentrate':ti,ab,kw) AND ('periapical surgery':ti,ab,kw OR 'apical surgery':ti,ab,kw OR 'endodontic surgery':ti,ab,kw OR 'endodontic microsurgery':ti,ab,kw OR apicoectomy:ti,ab,kw OR 'root-end surgery':ti,ab,kw OR 'periradicular surgery':ti,ab,kw) AND ('periapical lesion':ti,ab,kw OR 'apical periodontitis':ti,ab,kw OR 'bone healing':ti,ab,kw OR 'bone regeneration':ti,ab,kw OR CBCT:ti,ab,kw OR 'through-and-through defect':ti,ab,kw OR 'apicomarginal defect':ti,ab,kw)	15	28 April 2026

Supplementary Table S2

Study	Reason for exclusion
Govindaraju et al. (2023)	Non-comparative study involving a single patient, with no extractable data suitable for quantitative synthesis or meta-analysis.
Di Lauro et al. (2023)	Did not provide independent primary clinical data. The publication was used only as a secondary source for reference screening and was not eligible for qualitative or quantitative synthesis.
Qurban et al. (2024)	Non-comparative single-case report that did not meet the eligibility criteria for prospective comparative clinical studies with a control group.
Leopoldo et al. (2024)	Non-comparative case series with heterogeneous etiologies and no control group, precluding estimation of the effect attributable to PRF.
Rebimbas Guerreiro et al. (2025)	Scoping review without independent primary clinical data. The publication was used for background information but was not eligible for the effectiveness synthesis.
Krishnamurthy et al. (2025)	Non-comparative study including only two patients, with no comparator group and no data suitable for quantitative or qualitative comparative synthesis.

Govindaraju L, Antony DP, Pradeep S. Surgical management of radicular cyst with the application of a natural platelet concentrate: a case report. Cureus. 2023;15(1):e33992.

di Lauro AE, Valletta A, Aliberti A, Cangiano M, Dolce P, Sammartino G, Gasparro R. The Effectiveness of Autologous Platelet Concentrates in the Clinical and Radiographic Healing after Endodontic Surgery: A Systematic Review. Materials (Basel). 2023 Nov 16;16(22):7187.

Qurban HA, Hamadallah HH, Madkhaly MM, Hakeem MM, Alqutaibi AY. Leukocyte-Platelet-Rich Fibrin in bone regeneration after periapical surgery: a 30-month follow-up clinical report. Reports. 2024;7:32.

Leopoldo HA, Lopes MMP, Heggendorn FL. Association of the L-PRF technique in apicoectomies of different etiopathogenies. Rev Caribeña Cienc Soc. 2024;13(6):e4036.

Rebimbas Guerreiro S, Marto CM, Paula A, Paulo S. Platelet-rich plasma and platelet-rich fibrin in endodontics: a scoping review. Int J Mol Sci. 2025;26:5479.

Krishnamurthy M, Narayanan S, Leburu A, Selvendran KE, Praveen N. Application of titanium-platelet-rich fibrin (T-PRF) in the surgical management of a periapical lesion: a report of two cases. J Conserv Dent Endod. 2025;28:1302-1307.
